# Supplementary material for: Mesocrystal growth through oriented sliding and attachment of nanoplates
Source: Nat Commun. 2025 Dec 15;16:11240. doi: 10.1038/s41467-025-64852-7 (PMC12717213; doi:10.1038/s41467-025-64852-7)
Supplement: Supplementary file 1 — Supplementary Information [file 41467_2025_64852_MOESM1_ESM.pdf]

Supplementary Information

Mesocrystal growth through oriented sliding and attachment of nanoplates

Xiaoxu Li,[1] Tuan A. Ho[2], Honghu Zhang[3], Lili Liu[1], Ruipeng Li[3], Ping Chen[1], Mark E. Bowden[4], Sebastian T Mergelsberg[1], Hongyou Fan[2], James J. De Yoreo[1], Carolyn I. Pearce[5], Kevin M. Rosso[1], and Xin Zhang[1*]*

[1]Physical and Computational Sciences Directorate, Pacific Northwest National Laboratory, Richland, Washington 99352, United States

[2]Geochemistry Department, Sandia National Laboratories, Albuquerque, New Mexico 87185, United States

[3]National Synchrotron Light Source-II, Brookhaven National Laboratory, Upton, New York 11973, United States

[4]Institute for Integrated Catalysis, Pacific Northwest National Laboratory, Richland, WA 99352 United States

[5]Energy and Environment Directorate, Pacific Northwest National Laboratory, Richland, Washington 99352, United States

Corresponding authors: xin.zhang@pnnl.gov (XZ); kevin.rosso@pnnl.gov (KM)

Supplementary figures

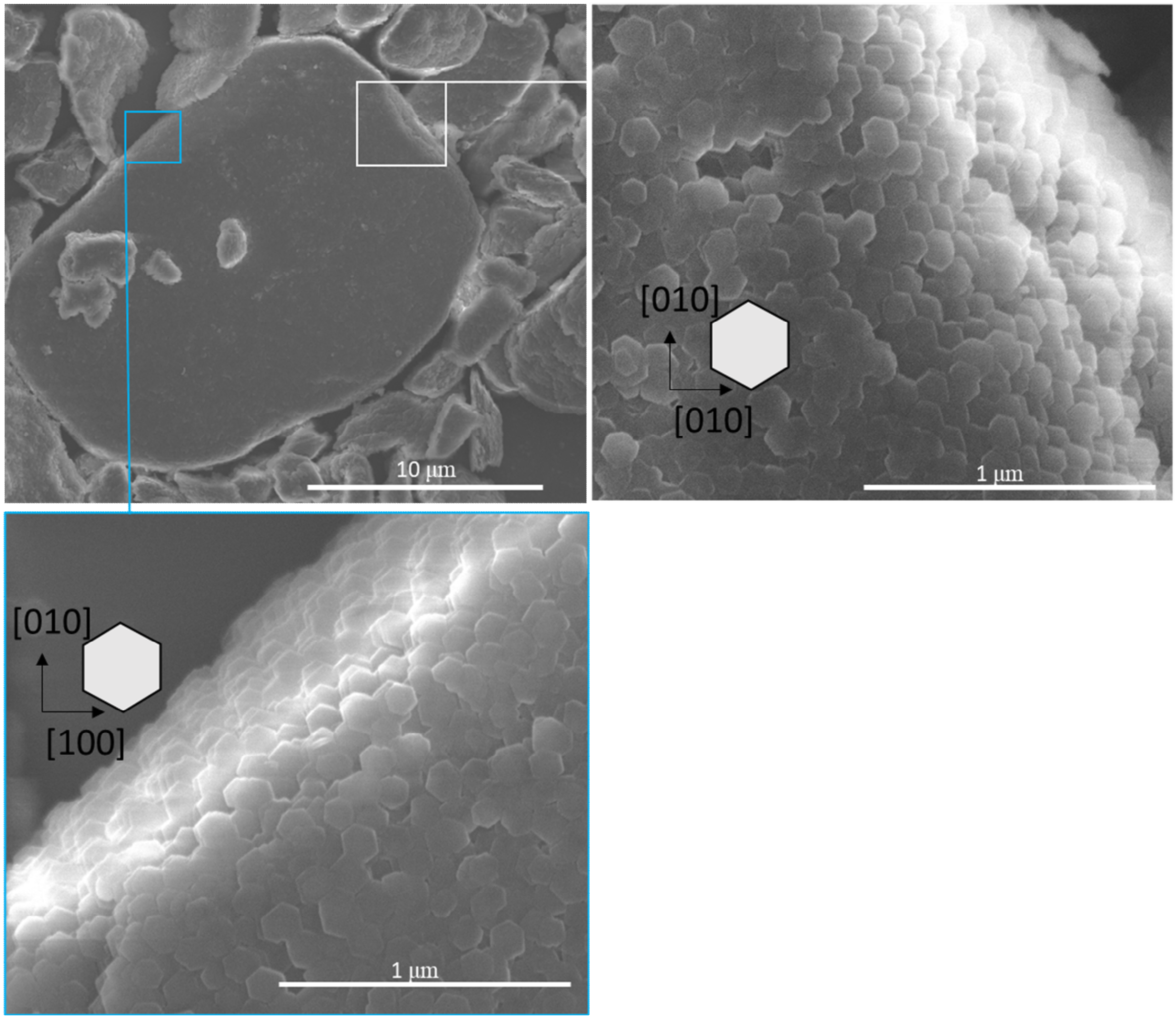

Supplementary Figure 1. **SEM images of the gibbsite mesocrystal**. The inserted hexagonal shapes mark the crystallography orientation of gibbsite nanoplates.

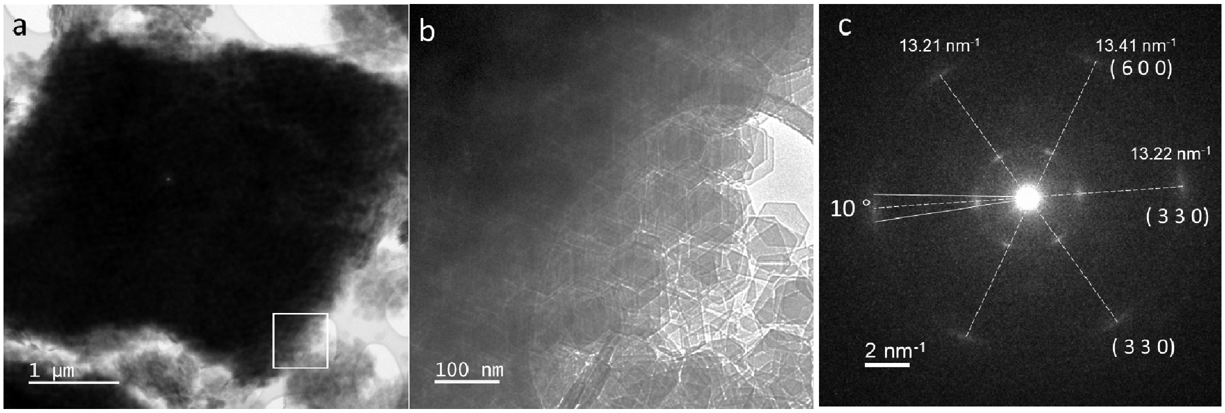

Supplementary Figure 2. **TEM characterization of gibbsite mesocrystal**. (a) The TEM image. (b) The zoomed-in TEM image of the white box in (a). (c) The selective area electron diffraction of (a) shows the diffraction pattern with the gibbsite (001) zone axis. The hexagonal superlattice structure doesn't show in TEM images.

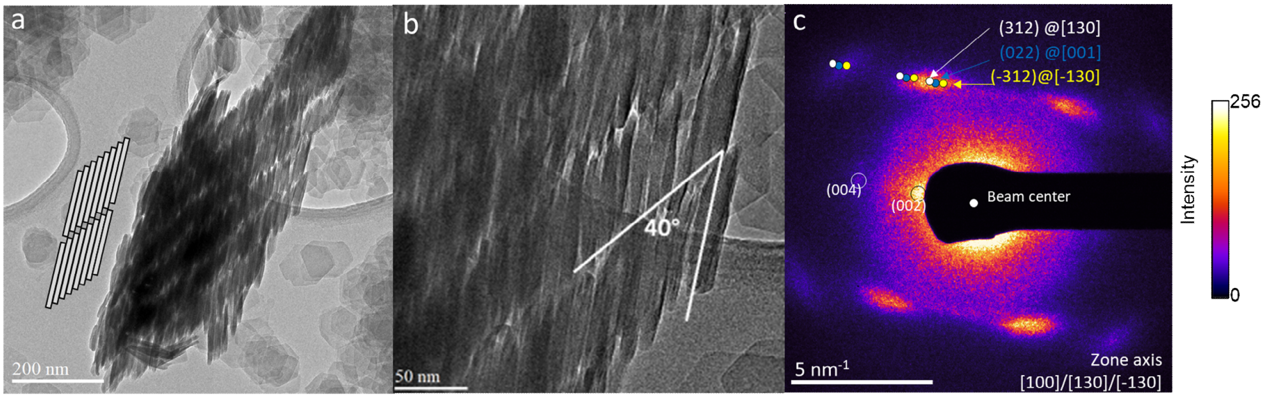

Supplementary Figure 3. **TEM characterization of the gibbsite mesocrystal from the side view.** (a) The TEM image. (b) The zoomed-in TEM image. (c) The selective area electron beam diffraction of the mesocrystal in (a). The indexing of the diffraction pattern shows the coexistence of gibbsite [100], [130], and [-130] zone axes. Color scale represents intensity in a linear scale.

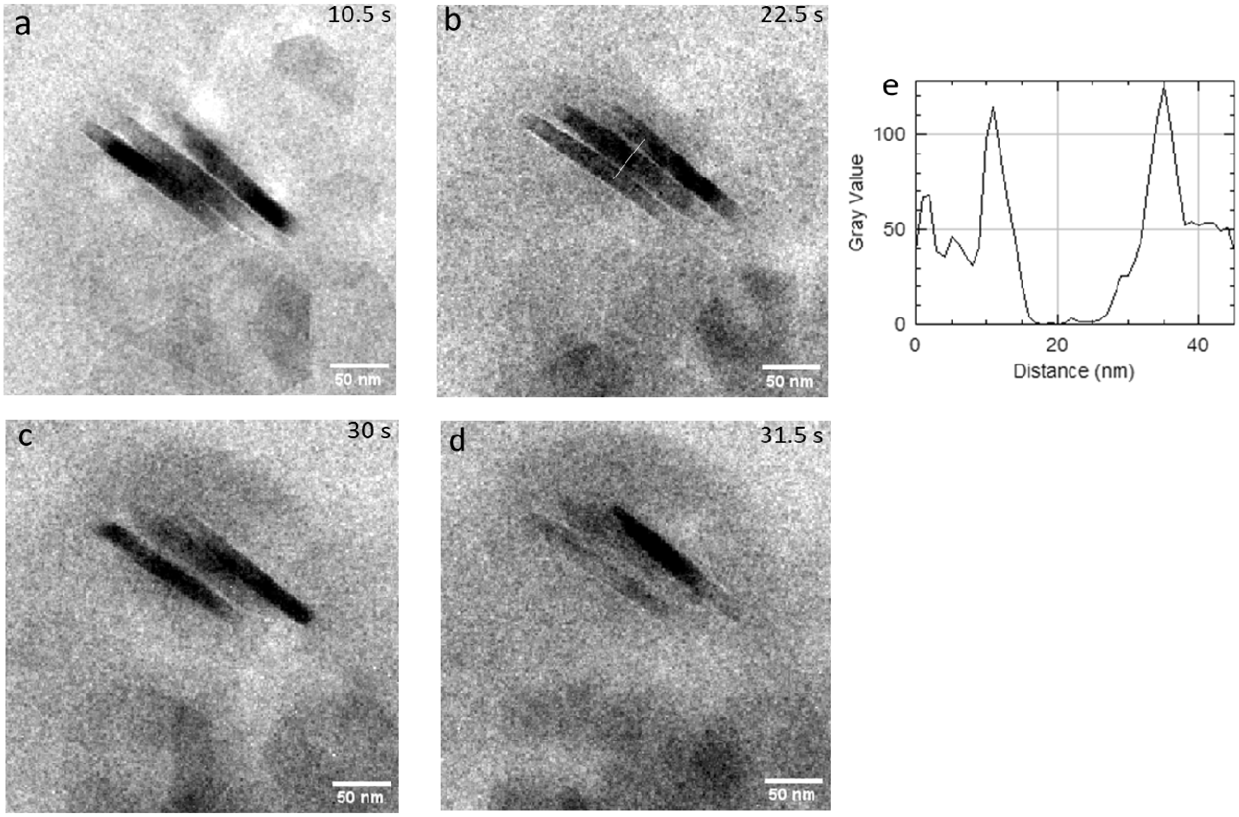

Supplementary Figure 4. **Separation of gibbsite nanoplate stacks observed by LC-TEM**. (a–d) Time-sequenced LC-TEM images showing the rotation and stacking behavior of gibbsite nanoplates in deionized water, extracted from Supplementary Movie 3. (e) Grayscale intensity profile across the nanoplate stack in panel (b) (where 0 corresponds to black and 256 to white).

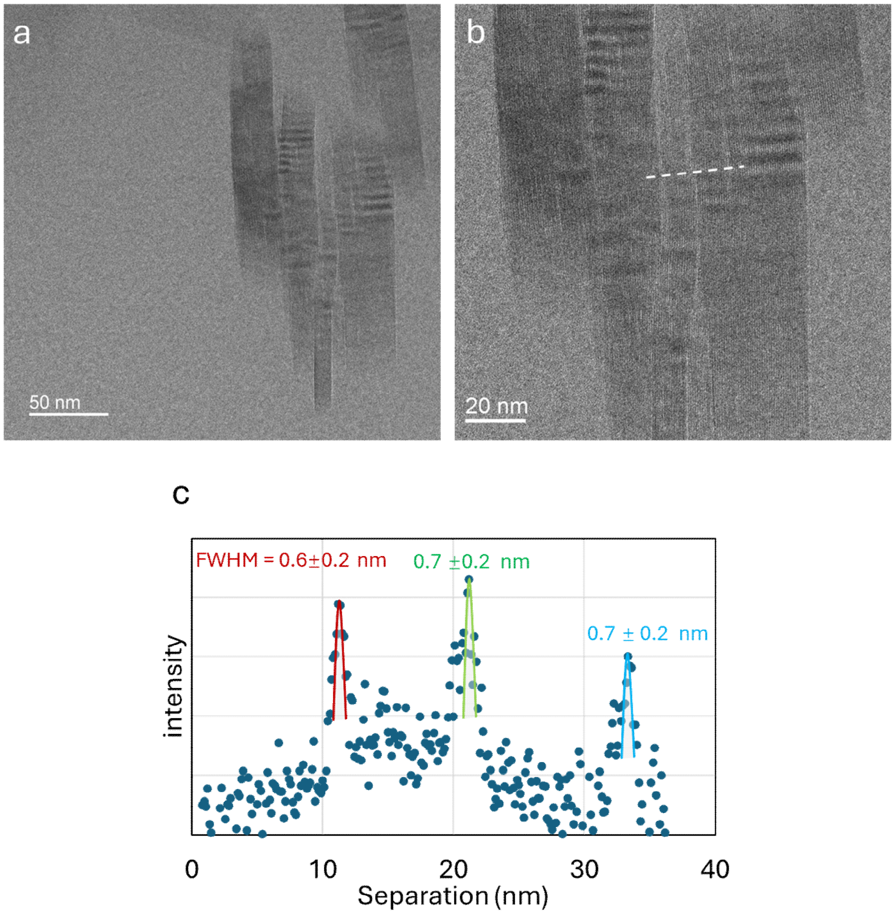

Supplementary Figure 5. **Separation of gibbsite nanoplates in a stack observed by high-resolution LC-TEM**. (a, b) Low- and high-magnification TEM images of stacked gibbsite nanoplates. (c) Intensity profile across a nanoplate stack, showing periodic peaks corresponding to inter-plate separations. Source data are provided as a Source Data file.

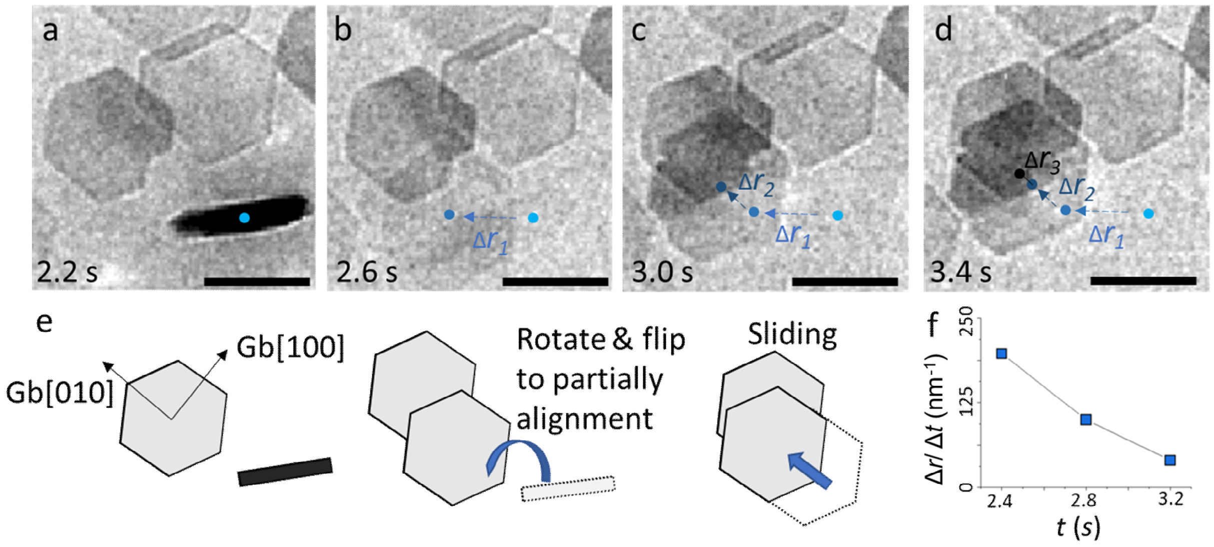

Supplementary Figure 6. ***In situ* liquid-cell TEM observation of gibbsite nanoplate OA from Supplementary movie 2**. (a–d) Time-lapse TEM images; scale bar: 100 nm. (e) Schematic illustration of the OA process and relative motions of the nanoplates. (f) Average 2D projected translational velocity ($\Delta r/\Delta t$) of the moving nanoplate during OA. The first data point ($t \approx 2.4$ s) corresponds to Δr_1 in panel (b) and includes both translational and rotational contributions during the flip–contact step; therefore, it is not directly comparable to the subsequent two points, which represent primarily lateral sliding. Nevertheless, the latter two points clearly show a decrease in sliding speed with increasing overlap area, consistent with Fig. 3. Source data of panel (f) are provided as a Source Data file.

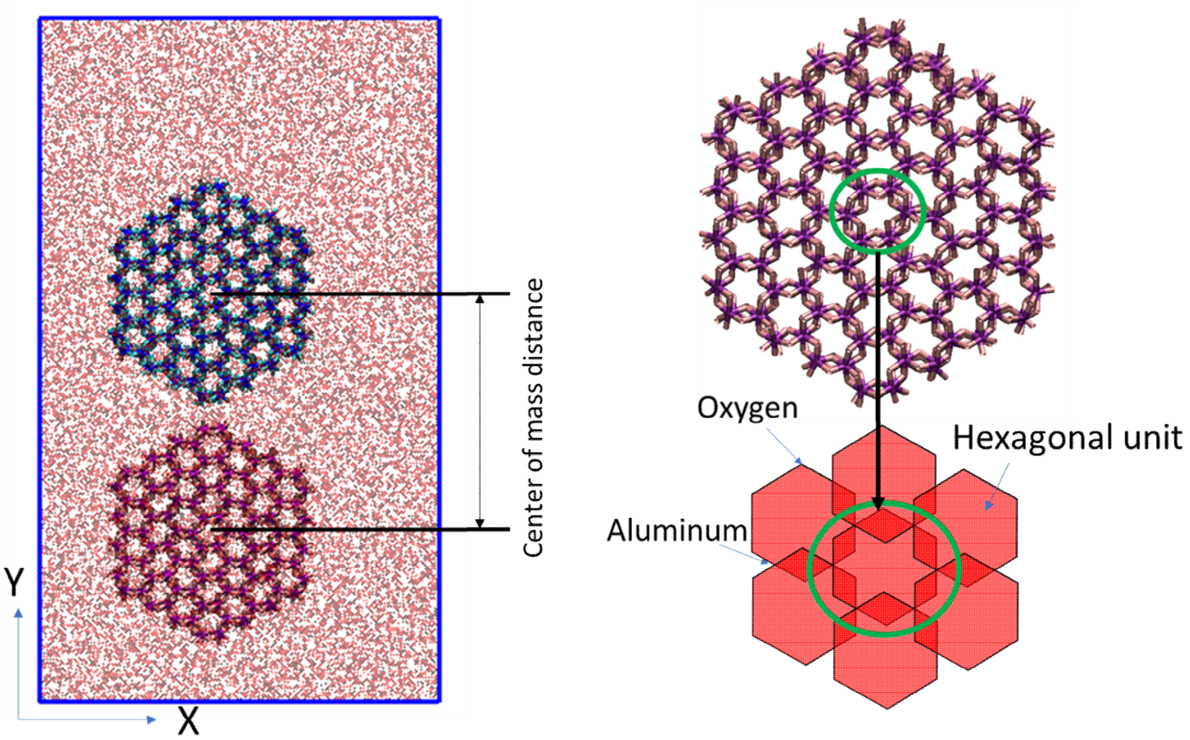

Supplementary Figure 7. **Snapshots of the molecular simulation.** Left: simulation snapshot demonstrating the system including 2 gibbsite particles that are 42 Å apart in the y direction. The simulation box size is 70×120×70.24 $Å^3$. Right: the basal surface can be viewed as an assembled repetition of many hexagonal units (bottom right) with oxygen atoms at a vertex and aluminum atoms located at the midpoint of an edge.

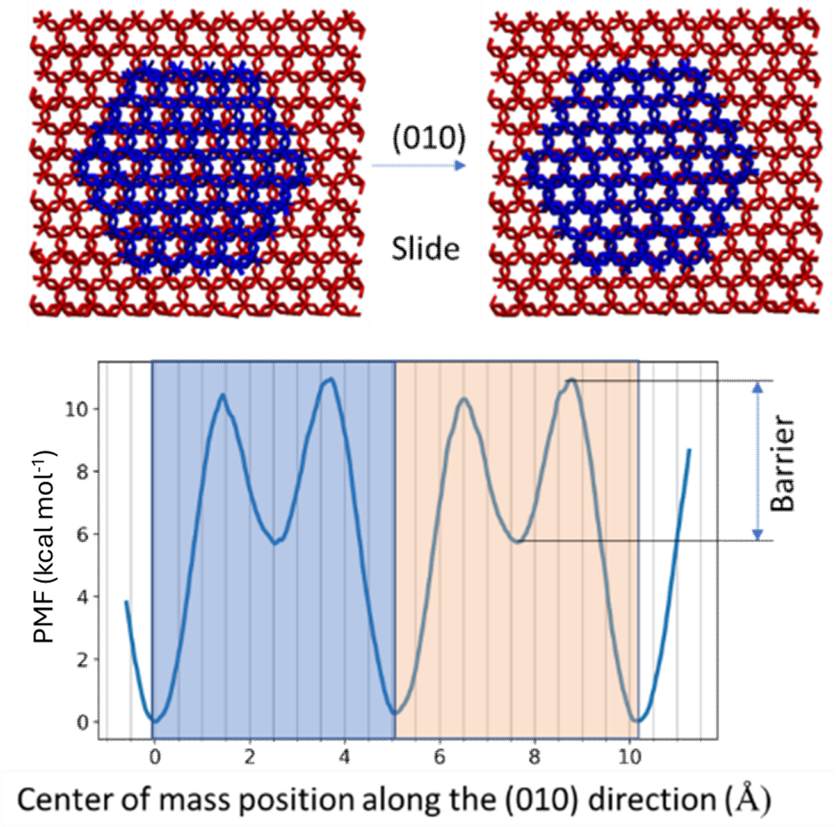

Supplementary Figure 8. **Simulation system and PMF results from our previous work**[1]. A particle (blue) slides on a gibbsite surface (red) in water along the (010) direction. The particle and surface are separated by a water layer (water is not shown). The shaded area indicates the periodicity of the PMF profile. Source data are provided as a Source Data file.

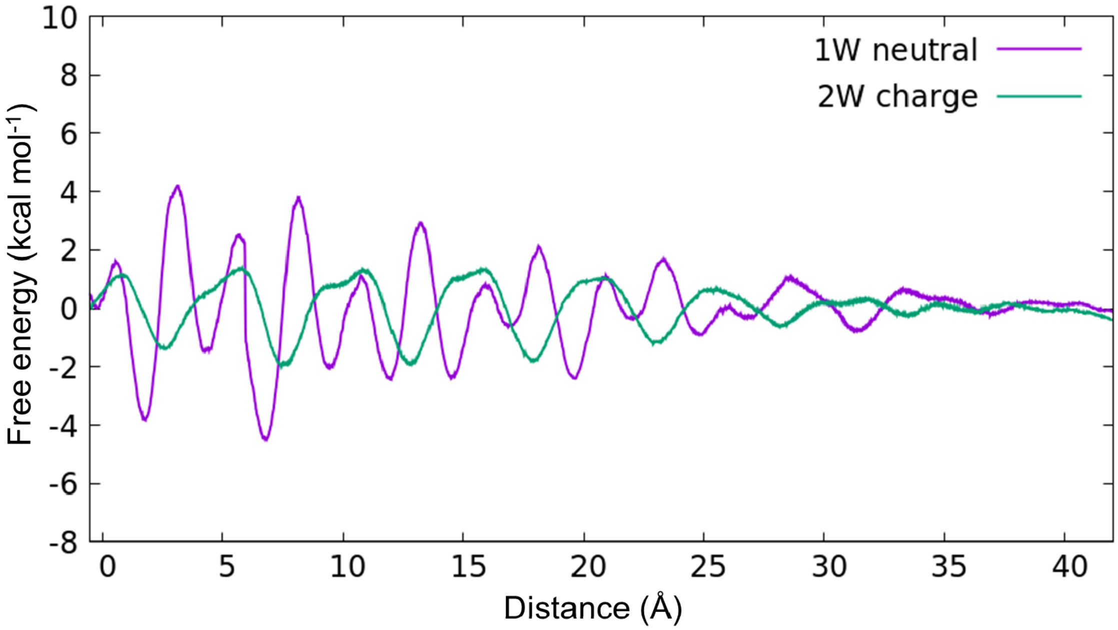

Supplementary Figure 9. **Potential of mean force (PMF) as a function of the center-of-mass distance between two particles during sliding motions along the [010] direction (i.e., overlap increases to the left).** The purple line shows the PMF obtained by sliding one particle relative to the other while maintaining a single water-layer separation in the z-direction, with both particles electrically neutral. The green line shows the PMF obtained by sliding one particle relative to the other while maintaining two water layers between the particles; in this scenario, both particles have positively charged edge surfaces with a charge density of +0.1897 C/m². The charge is evenly distributed on the hydrogen atoms of the edge hydroxyl groups. Cl^- ions are introduced to balance these positive surface charges in the simulation, ensuring overall charge neutrality. Source data are provided as a Source Data file.

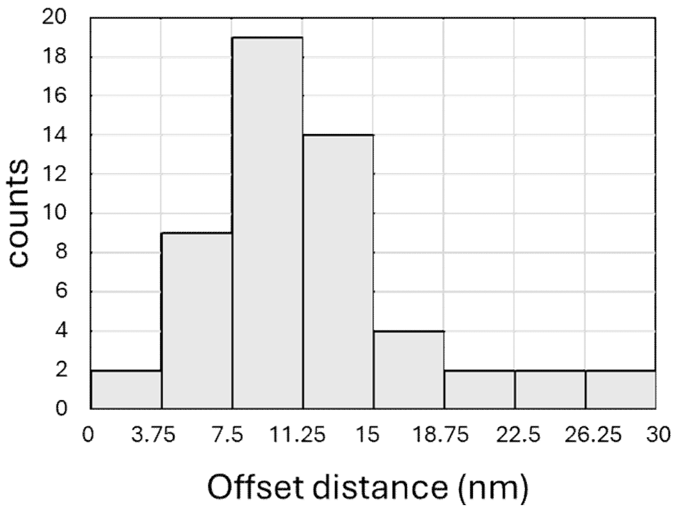

Supplementary Figure 10. **Distribution of offset distance of adjacent gibbsite nanoplates in the stacks measured from TEM and SEM images** (n=50). Source data are provided as a Source Data file.

Supplementary reference

1 Vu, T. V., Ho, T. A. & Criscenti, L. J. Roles of Hydrogen Bonds and Alignment in Oriented Attachment of Gibbsite Nanoparticles: Insights from Molecular Dynamics. J. Phys. Chem. C **127**, 8695-8703 (2023). https://doi.org:10.1021/acs.jpcc.2c08157
