# Supplementary material for: Mesocrystal growth through oriented sliding and attachment of nanoplates
Source: Nat Commun. 2025 Dec 15;16:11240. doi: 10.1038/s41467-025-64852-7 (PMC12717213; doi:10.1038/s41467-025-64852-7)
Supplement: Supplementary file 2 — Description of Additional Supplementary Files [file 41467_2025_64852_MOESM2_ESM.pdf]

Description of Additional Supplementary Files

File name: Supplementary Movie 1

Description: In situ liquid-cell TEM observation of the OA of gibbsite nanoplates (movie 1).

File name: Supplementary Movie 2

Description: In situ liquid-cell TEM observation of the OA of gibbsite nanoplates (movie 2).

File name: Supplementary Movie 3

Description: In situ liquid-cell TEM observation of the gibbsite nanoplate stack from a side view.
